# Mismatch Between Birth Date and Vegetation Phenology Slows the Demography of Roe Deer

**DOI:** 10.1371/journal.pbio.1001828

**Published:** 2014-04-01

**Authors:** Floriane Plard, Jean-Michel Gaillard, Tim Coulson, A. J. Mark Hewison, Daniel Delorme, Claude Warnant, Christophe Bonenfant

**Affiliations:** 1 Laboratoire “Biométrie et Biologie Évolutive,” Unité Mixte de Recherche 5558, Université Claude Bernard Lyon 1, Lyon, France; 2 Department of Zoology, The Tinbergen Building, University of Oxford, Oxford, United Kingdom; 3 INRA, UR035 CEFS, B.P. 52627, Castanet-Tolosan cedex, France; 4 Centre National d'Études et de Recherches Appliquées Cervidés-Sangliers, Office National de la Chasse et de la Faune Sauvage, Paris, France; University College London, United Kingdom

## Abstract

Marked impacts of climate change on biodiversity have frequently been demonstrated, including temperature-related shifts in phenology and life-history traits. One potential major impact of climate change is the modification of synchronization between the phenology of different trophic levels. High phenotypic plasticity in laying date has allowed many bird species to track the increasingly early springs resulting from recent environmental change, but although changes in the timing of reproduction have been well studied in birds, these questions have only recently been addressed in mammals. To track peak resource availability, large herbivores like roe deer, with a widespread distribution across Europe, should also modify their life-history schedule in response to changes in vegetation phenology over time. In this study, we analysed the influence of climate change on the timing of roe deer births and the consequences for population demography and individual fitness. Our study provides a rare quantification of the demographic costs associated with the failure of a species to modify its phenology in response to a changing world. Given these fitness costs, the lack of response of roe deer birth dates to match the increasingly earlier onset of spring is in stark contrast with the marked phenotypic responses to climate change reported in many other mammals. We suggest that the lack of phenotypic plasticity in birth timing in roe deer is linked to its inability to track environmental cues of variation in resource availability for the timing of parturition.

## Abstract 2

This study of a French deer population reveals the demographic costs associated with the failure of a herbivore to modify its life cycle timing to respond to a warming world.

## Author Summary

Climate change can alter the synchronization of life cycles between organisms at different points in the food chain. If species do not respond to climate change, the timing of peak resource availability may fail to match the timing of peak energy expenditure. Many bird species have been able to advance their laying date to match a change in the timing of caterpillar abundance. Herbivores are similarly expected to track changes in the timing of vegetation growth. In this study, we combine statistical analysis with demographic modeling to analyze the influence of a climate-driven shift in the timing of the spring vegetation flush on the birth date and demography of roe deer. In recent years, climate change has generated a marked increase in local temperatures and a progressively earlier vegetation flush. Despite these changes, we observed no shift in timing of the birth date of roe deer over the 27-year study period. This failure to track environmental change resulted in a mismatch between vegetation flush and birth date, which in turn caused a decrease in survival of the young, and hence a reduction in roe deer fitness. Birth date was under strong directional selection, but was not strongly heritable, suggesting that any evolutionary response of birth date to climate change might be limited. We suggest that a plastic response in birth date did not occur because reproduction is triggered by day length rather than resource availability in roe deer.

## Introduction

Marked impacts of climate change on biodiversity have frequently been demonstrated, including temperature-related shifts in phenology and life-history traits [1]. Species living at high altitudes or latitudes are particularly affected by climate change [2],[3], but widespread species inhabiting temperate areas are also responding [4]. Global temperatures have risen by 0.89°C since 1901 [5], and this has led to an advance in the timing of key life-history events by, on average, 2.8 d per decade [6]. Earlier springs have caused phenological modifications in most taxonomic groups [1],[7]. The phenology of vegetation, particularly trees, has advanced with time (by 3.3 d per decade [6]). A failure of species to track these changes may have important demographic consequences that, in turn, could impact conservation and management issues. Changes in the timing of reproduction have been well studied in birds [8]–[10], but have only recently been considered in mammals [3],[4],[11]. These studies suggest that a change in the timing of peak resource availability typically generates a change in median laying or breeding date [9],[12],[13]. This response ensures that individuals can synchronize their energetic demands for offspring production and provisioning [14] with the period when environmental conditions are the most favorable [15].

A key question about the consequences of global change is which species can respond and how [16]. Although most studied species have responded to match resource availability with energy requirements, the response is not always exact or immediate [17],[18]. Phenotypic plasticity may play a major role in the adjustment of reproductive timing to earlier springs [19]. In a population of great tits, those individuals that could vary their reproductive timing the most had higher fitness [20]. But for individuals to shift their reproductive cycle in order to track environmental modifications, they require a reliable environmental cue [21],[22]. The timing of breeding is influenced by photoperiod in many species of birds and mammals [23]–[25], a cue that is clearly unaffected by climate change. Nonetheless, some species, including the great tit and red deer, rely on temperature [26],[27] to minimize the mismatch between birth timing and the peak resource availability. If earlier breeding increases fitness, selection could also drive a micro-evolutionary change in terms of advanced reproductive timing [8] as long as birth date is heritable. The relative role of phenotypic plasticity and micro-evolutionary change remains largely unquantified [28], although Réale et al. [4] showed that the advance in birth timing in red squirrel was mostly due to phenotypic plasticity rather than micro-evolution. Although some species have advanced their birth timing in response to increasing temperature, some species have not [29] whereas others have delayed their reproductive phenology [1]. For instance, Columbian ground squirrels have delayed their breeding phenology by 0.47 d per year over a period of 20 y, leading to a reduction in fitness by a half between 1993 and 2003 [3]. On the other hand, birth timing in caribou advanced at a much slower rate than the vegetation flush over a period of 33 years, so that the mismatch between birth timing and peak resource availability increased, causing calf production to decline [30],[31].

Births are highly seasonal and synchronous in most large herbivores [32], including roe deer [33],[34], in which more than 90% occur within 1 mo [35]. Roe deer females are income breeders and selectively feed on highly digestible and nutritious young shoots, especially during early lactation when energetic demand peaks [14]. Synchrony between births and the peak availability of high-quality vegetation is expected to be crucial for successful recruitment. The reproductive cycle of roe deer is unique among ungulates, including a phase of embryonic diapause that appears not to vary in duration among females [36]. As in reindeer where reproductive timing may be driven by day length [37], both ovulation and conception dates appear to be under the control of photoperiod [38], which could explain the lack of variation in parturition date across years for a given female [33],[35].

Focusing on the mismatch between birth date and plant phenology, we investigate how climate change is currently affecting roe deer fitness. Our study on the intensively monitored roe deer population at Trois Fontaines, eastern France, spans 27 y, from 1985 to 2011. We tested the three following predictions: (i) As most mammals studied so far have shown a response to climate change, we expected that roe deer births should occur earlier in response to the advance in vegetation phenology. (ii) Because parturition date varies little across years for a given roe deer female [35], indicating limited phenotypic plasticity, but because it has been shown to be heritable in mammals [39],[40], and markedly influences early offspring survival [40], we expected any change in birth timing to be mainly the result of natural selection. And (iii) because such micro-evolutionary responses are often delayed, we expected that despite any advance in birth timing, the mismatch between peak energetic demand and peak resource availability should likely increase over time, leading to negative impacts on roe deer performance. Our study provides a unique quantification, to our knowledge, of the demographic costs associated with the failure of a species to modify its phenology in response to a warming world.

## Results

### Climate Change and Birth Date

**Figure 1:**
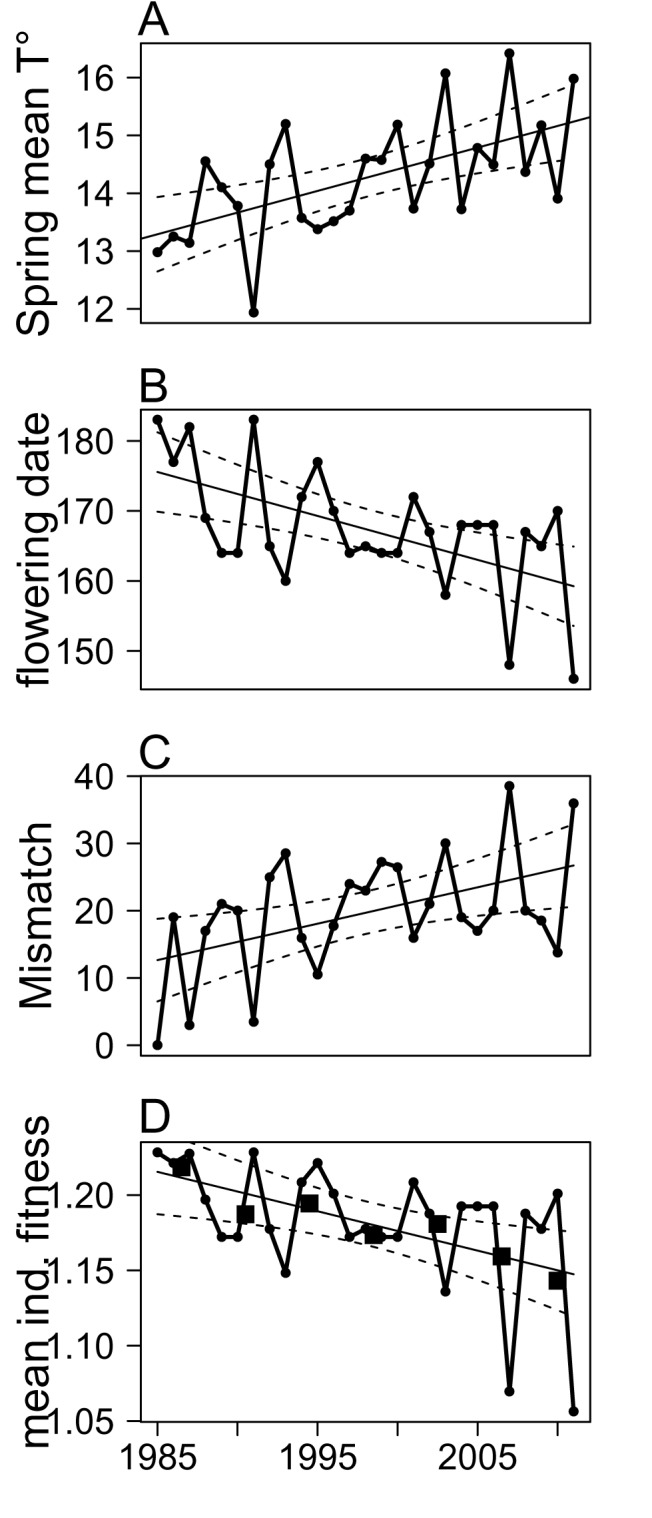
Temporal variation in spring temperature (A), flowering date in the vineyards of the Champagne region (B), the observed population mean of the mismatch between flowering date and median parturition date (C), and mean individual fitness predicted from the IPM (D) in the roe deer population of Trois Fontaines, France from 1985 to 2011. The mismatch was estimated as the difference between median birth date and annual flowering date in the Champagne vineyards. We standardized this measure (by subtracting the observed value of the mismatch in the first year of study (1985) from this variable) to obtain a relative measure of mismatch ranging from 0 in 1985 to 36 d in 2011. Predicted trends are presented as black lines with 95% confidence intervals (dashed lines). Geometric means of mean individual fitness were calculated over periods of 4 y (black squares).

Analysis of local weather variables revealed a strong local impact of climate change that translated into increasingly earlier and warmer springs over time. Annual spring (April to June) temperature increased by 0.07°C per year (*SE* = 0.02, *p* = 0.001, Figure 1) at Trois Fontaines over the study period. Analysis of flowering date in the vineyards of the Champagne region indicated that annual timing of plant phenology in the region had advanced by 0.6 d per year (*SE* = 0.18, *p* = 0.002, Figure 1) over this period, so that the peak in availability of high-quality resources for roe deer was increasingly early from 1985 to 2011. Spring mean temperature and flowering date in the Champagne region were negatively correlated over this period (*ρ* = −0.89, *p*<0.001). We therefore used flowering date as a proxy of the vegetation flush and compared it to annual variation in the timing of roe deer births. Roe deer give birth about 1 mo before the onset of flowering in Champagne because they preferentially feed on leaves and young shoots that become available before flowering. The mismatch between median birth date and vegetation phenology was estimated from the difference between median birth date and annual flowering date in the Champagne vineyards. We standardized this measure (by subtracting the observed value of mismatch in the first year (1985) from this variable) to obtain a relative measure of mismatch ranging from 0 in 1985 to 36 d in 2011.

In contradiction with our first prediction, annual median birth date did not occur earlier over time. Both the mean and median birth dates of roe deer at Trois Fontaines remained remarkably stable among years (based on 1,095 birth dates, *mean* = 136.1, *SE* = 8.56; time trend, *t* = −0.82, *p* = 0.421; and median = 136, the 16th of May, *t* = −1.23, *p* = 0.232 for mean and median birth dates, respectively, Figure S4A). Neither the spring mean temperature nor flowering date in the Champagne region had a detectable influence on median birth date (Pearson's product moment, *ρ* = −0.07, *p* = 0.730 and *ρ* = 0.11, *p* = 0.575 for spring mean temperature and flowering date, respectively). Consequently, the mismatch between median birth date and vegetation phenology increased by 0.54 d per year (*SE* = 0.20, *p* = 0.011, Figure 1) between 1985 and 2011. We did not find any correlation between median birth date and other environmental drivers (Table S1), suggesting that roe deer females are unable to track these potential environmental cues.

### Parturition Date, a Trait Under Strong Selection Pressure

We investigated whether birth date of roe deer fulfilled the three necessary conditions for evolutionary change to occur: variability, heritability, and a selection pressure [41]. First, variation in parturition date among roe deer females has been recently quantified and found to be consistently high within several populations [35], with long-lived and/or heavier females (i.e., high-quality individuals) giving birth earlier than low-quality females [42]. Second, we found no strong statistical support for heritability in parturition date when estimated from the parent–offspring relationship based on 28 daughter–mother pairs (*β* = 0.234, *SE* = 0.13, *p* = 0.094, *h* ^2^ = 0.127). Third, we identified directional selection favoring early births, with a strong negative relationship between individual birth date and individual early survival from May 12th onwards (on a logit scale *β* = −0.06, *SE* = 0.01, *p*<0.001, Figure S1). Note that year was included as a categorical variable in this model to control for interannual variation in environmental conditions affecting early survival. A model including a threshold effect of individual birth date on individual early survival provided a better fit than a linear (Δ*AIC* = 6.12) or a quadratic (Δ*AIC* = 0.50) model. Thus, a fawn born before May 12th had, on average, a 50% chance of surviving to 8 mo of age, whereas a fawn born on May 31st had, on average, only a 24% chance of surviving to that age. Adding a term describing interactive effects between birth date and year did not improve the fit of the model (Δ*AIC* = 9.28), indicating that the response of individual early survival to individual birth date was consistent across cohorts. Taken together these results suggest that we should not expect a strong micro-evolutionary response of parturition date in roe deer.

### Demographic Consequences

**Figure 2:**
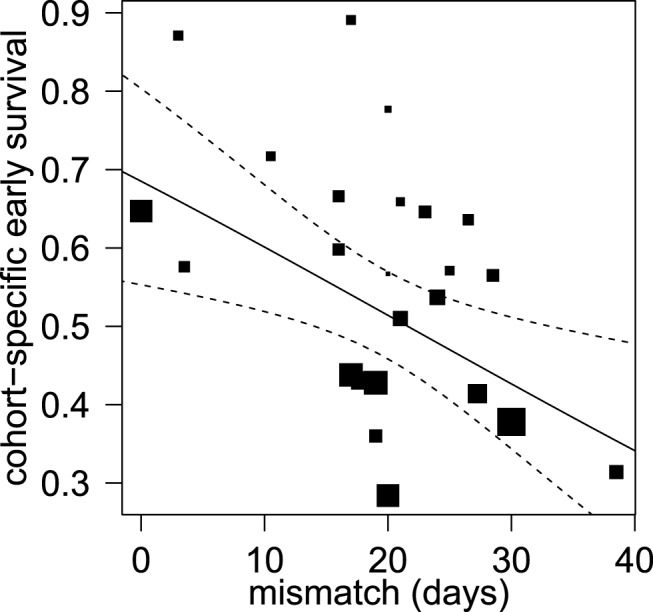
Variation in mean cohort-specific early survival in relation to the mismatch between annual median birth date and vegetation phenology in the roe deer population of Trois Fontaines, France. The predicted relationship is represented by a black line with 95% confidence intervals (dashed lines). Data used to fit the models are represented by black squares whose size is proportional to the standard error of mean cohort-specific early survival.

At the population level, cohort-specific survival (measured as the proportion of fawns that survived to the onset of winter each year) was negatively correlated with our index of mismatch (arcsine-square root transformation: *β* = −0.009, *SE* = 0.003, *p* = 0.012). Adding a quadratic term (Δ*AIC* = 1.95) or a threshold effect (Δ*AIC* = 1.86) of mismatch did not improve model fit. Early cohort-specific survival of juveniles decreased by 40% with an increase in mismatch of 1 mo (Figure 2).

**Figure 3:**
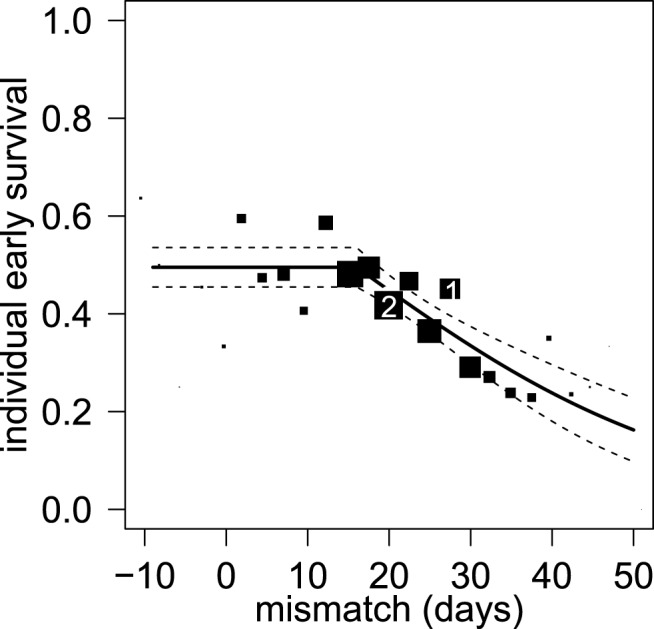
Influence of the mismatch (measured as the difference in days between individual birth date and flowering date in Champagne) on individual early survival in the roe deer population of Trois Fontaines, France. The predicted values are presented after back-transformation from a logit scale with 95% confidence intervals (dashed lines). Data used to fit the model are represented by black squares whose size is proportional to the number of observed births within periods of 2.5 d (examples in the figures: 1 (in white), 82 fawns; 2 (in white), 115 fawns).

At the individual level, the mismatch was a better predictor of early survival (*R* ^2^ = 0.037) than birth date (*R* ^2^ = 0.025) (Δ*AIC* = 8.90, Table S2; note that year was not included in these models because interannual variation was integrated within the mismatch variable). Individual early survival was constant when the mismatch was 16 d or less, but then decreased linearly beyond 16 d of mismatch (Table S2, Figure 3). When birth occurred at least 1 mo (35 d) before flowering date in Champagne's vineyards, a fawn had an expected probability of 0.5 of surviving to 8 mo of age, whereas this probability was only 0.25 when birth occurred 2 wk prior to flowering (Figure 3).

We built an IPM describing the temporal dynamics of parturition date in our roe deer population to quantify the impact of this increasing mismatch on roe deer fitness. The distribution of parturition date in the population at time *t*+1 depends on the distribution of parturition date at time *t* and on the four relationships linking parturition date with survival, recruitment, transition between two successive parturition events, and inheritance of parturition date between mother and offspring (see Text S1, Tables S2, S3, S4, and Figure S2 for further details [43],[44]). Annual flowering date in the vineyards of the Champagne region was included in the IPM to model the local shift in plant phenology. As most roe deer females give birth to two fawns [45] and the sex ratio is close to 0.5 at birth at Trois Fontaines [46], the recruitment function linking the number of female offspring a mother has successfully weaned given its parturition date was modeled by individual early survival (Table S5, Figure 3).

The IPM predicted mean parturition date to occur on the 17th of May each year, with a very slight, but statistically significant, advance of just 0.27 d over the whole 27-y study period (*β* = −0.010, *SE* = 0.003, *p* = 0.006, Figure S4B). The model also predicted earlier parturition as females aged, with 2-y-old females giving birth, on average, 5 d later than older females (Figure S5). The estimated population growth rate, and so the mean fitness of females in the population, decreased by 6% on average over the study period (*β* = −0.003, *SE* = 0.001, *p* = 0.008, Figure 1), from 1.23 in 1985 to 1.06 in 2011. Marked variability in environmental conditions between successive years often leads to a decrease in the arithmetic mean population growth rate [47]. Consequently, we estimated the geometric mean of population growth rate for successive periods of 4 y. The geometric mean population growth rate also decreased over the study period (*β* = −0.009, *SE* = 0.002, *p* = 0.002). Thus, the IPM allowed us to demonstrate a clear impact of the mismatch between energy demand and peak resource availability on mean fitness, which declined in this population of roe deer over the entire study period. In accordance with these results, we also observed a decrease in mean annual female reproductive success over the study period (*β* = −0.027, *SE* = 0.005, *p*<0.001; note that these data are independent of those used to build the IPM, see Figure S3).

## Discussion

This study has demonstrated that mean fitness is currently decreasing in this roe deer population due to the lack of response in parturition date to the increasingly early availability of high-quality resources induced by climate change. Warming at Trois Fontaines over the last 27 y (0.46°C per decade) was more than threefold greater than the average global expectation from the 2013 IPCC report on climate change (0.12 [0.08–0.14] per decade since 1951, [5]). This local warming has led to an advance in spring plant phenology [48] demonstrated by the advance in flowering date in Champagne's vineyards. In contrast to most other studied mammals that have been able to track resource availability by advancing their birth timing [3],[4],[13],[49], the median birth date of roe deer remained constant over years. This generated an increased mismatch between mean birth date and phenology of the vegetation such that at the end of the study period fawns were born relatively later with respect to the peak in availability of high quality resources. Post and Forchhammer [30] were the first to describe a negative impact of a mismatch between resource availability and birth timing on calf production in Greenland caribou. In our study, climate change over recent decades has had a similarly negative impact on early survival (i.e., a “climatic debt” [16]), both at the individual and at the population levels. Furthermore, we were able to show that this mismatch between parturition and the availability of highly digestible forage led to a decline in mean fitness of 6% over the study period, and of 14% between 1985 and those years when the vegetation flush was particularly early (2007 and 2011). This link between plant phenology and roe deer population dynamics, mostly driven through recruitment, is the likely mechanism for the observed decrease in population growth rate over time [50].

Our study provides an illustration of the probable fitness costs for species which do not respond to climate change. Indeed, the increasing mismatch between the peak of roe deer births and the onset of the vegetation flush in recent years had a negative impact on both early survival and mean fitness. Previous studies have reported an impact of climate change on recruitment [30],[51],[52]. However, the influence of mismatch on fitness and population dynamics has received much less attention. IPMs allow the phenotypic consequences of climate change to be explored (see also [49]), which is not possible using classical statistical methods. In contrast to a recent study on birds [53], we found that population growth rate of roe deer was not buffered against phenological mismatch. Mean fitness was most strongly affected during years when plant phenology was particularly early, for example, in 2007 and 2011 (*λ* = 1.07 versus *λ* = 1.23 for the first year of the study). We can therefore predict that this increasing mismatch will further increase the energetic costs of breeding for females [54] as spring phenology continues to advance in the future.

The lack of response in roe deer birth date to climate change provides a stark contrast with the previous findings on most mammalian species studied to date, which have shown phenotypic responses to climate change [3],[4],[13],[49]. Despite the clear selection pressure that we demonstrated, which should favor earlier births over time, we showed that a strong evolutionary change is not expected in roe deer. Indeed, we found no strong statistical support for heritability of birth date, despite the fact that parent–offspring regressions are known to overestimate heritabilities [55]. However, as the number of mother–daughter pairs (*N* = 28) available to assess heritability of birth date was low, further work on a much larger sample size is required to explore this question. Nonetheless, both the classical statistical approach and the IPM provided similar results and clearly indicated no change in roe deer reproductive timing. Although roe deer did not exhibit an evolutionary response to climate change, why have they not responded plastically [4],[20]?

Roe deer females appear unable to track environmental cues such as temperature to time their birth event. Birth timing in mammals is mainly driven by the date of conception and gestation length. Ovulation and, thereby, conception date is mainly under the control of photoperiod in roe deer [38]. Gestation begins with a phase of embryonic diapause that probably originally evolved to increase gestation length [56], but we expected this historical selection pressure for delayed birth to be counterbalanced by selection for earlier birth date in response to climate change over recent times. However, diapause appears to be triggered by an intrinsic mechanism involving the mother or even the embryo itself, and the 5-mo duration appears not to vary among females [36]. In many species, adaptive phenotypic plasticity has generated a response to changes in phenology (great tit [57], red squirrel [4]). In red deer, a species related to roe deer that is able to track earlier plant phenology [13], gestation length decreased with increasing average temperature in March [58]. In contrast, in roe deer, we have shown that parturition timing is independent of changes in temperature and in the onset of the vegetation flush, suggesting this lack of phenotypic plasticity in birth timing is associated with an inability to track environmental cues of variation in resource availability for the timing of parturition.

Earlier plant phenology is likely the main cause of the observed decrease in early survival, and thereby in mean fitness, in this roe deer population. Although the roe deer population consistently displayed positive growth over the 27-y study period (i.e., *λ* consistently higher than 1), population growth rate (and therefore average individual fitness) decreased in a continuous fashion by 6% over this period. Moreover, temperatures are expected to increase further in the future, causing the phenology of vegetation to advance still further. We suggest that these combined effects could impose a brake on the demographic and geographical expansion of roe deer, a common and previously successful species across all Europe [59].

## Materials and Methods

### Studied Site and Population

Trois Fontaines (48°43N, 2°61W) is an enclosed 1,360 ha forest located near Saint-Dizier, at the border of Marne and Haute-Marne counties in north-eastern France. In spring (from mid-March to mid-June), the number of rainy days averaged 31.5 (ranging between 18 and 52 d during the study period 1985–2011) and the temperature averaged 10.4°C (ranging between −6.06°C and 15.00°C). The forest is dominated by oak (*Quercus sp.*) and beech (*Fagus sylvatica*). Roe deer feed mainly on coppice and the understory is dominated by hornbeam (*Carpinus betulus*), ivy (*Hedera helix*), and bramble (*Rubus sp.*).

### Data Collection

The roe deer population at Trois Fontaines has been intensively monitored for more than 35 y by the Office National de la Chasse et de la Faune Sauvage based on a detailed Capture-Mark-Recapture program. Roe deer are individually marked using numbered collars and ear-tags. A systematic search for newborn fawns was conducted every year from late April to mid-June between 1985 and 2010 [60]. In 2011, searches ended earlier and the last fawn was found on May 20th. Fawns were handled by experienced people, ear-tagged, and weighed. Their sex was recorded and their age estimated to the nearest day using umbilicus characteristics and behaviour at marking [61]. Birth dates were back-calculated using these estimated ages and the day of capture. The average age at marking was about 5 d, and all fawns were marked within 20 d. The identity of the mother for a given fawn was established, when possible, through direct observations of lactating behavior or by the identification of an escaping female in the vicinity of the fawn. From January to March, annual capture sessions took place, with capture of more than 50% of the roe deer population each year, providing reliable information on the fate of animals marked at birth. Individual early survival was defined as the probability of survival of a fawn from birth to the next winter (see [62] for further details). At Trois Fontaines, fawn mortality was most likely associated with shortage of high-quality food because this population is not subject to marked predation or hunting pressure. At the population level, mean cohort-specific early survival was estimated using Capture-Recapture analyses [63] from 1985 to 2011. Cohort-specific early survival could not be estimated with accuracy for 2012 (see [62]), so these data were not included here. Data are available from Table S6.

### Climate Change and Parturition Date

The local daily temperature was collected from the Météo-France weather station of Saint-Dizier located at less than 5 km from the study site. Spring temperature (April to June) was used to assess the magnitude of climate warming at the local scale. To measure annual changes in vegetation phenology, we used the mean flowering date of vineyards in the Champagne region collected by the Comité Interprofessionnel du Vin de Champagne and available on the website of the French Observatoire National sur les Effets du Réchauffement Climatique (http://www.developpement-durable.gouv.fr/-Impacts-et-adaptation-ONERC-.html). Flowering date is little influenced by human activity and reliably reflects the phenology of the vegetation of that year. Moreover, flowering date in Champagne is highly correlated with the local sum of degree-days (*ρ* = −0.61, *p*<0.001), which is often used to index vegetation growth [13],[64], with spring temperature (*ρ* = −0.83, *p*<0.001) and with annual mean temperature (*ρ* = −0.57, *p*<0.001). As a consequence, flowering date in Champagne reliably indexes the overall changes in plant phenology over years. When flowering date in Champagne was early in the year, we assumed that the availability of high-quality resources for roe deer provided by spring vegetation was also early. Consequently, we used the difference between birth date and flowering date in Champagne as a measure of the mismatch between birth date and peak resource availability.

To assess temporal trends in local temperature, vegetation phenology, and birth date, we fitted linear regressions with a Gaussian error. Subsequently, we examined the relationships of cohort-specific median birth date with mean spring temperature and with flowering date in Champagne to test whether birth date tracked climate change. To quantify the mismatch between median birth date and the vegetation flush at the population level, we subtracted the median birth date from the annual flowering date.

### Birth Date: A Trait Under Selection?

Available data were not detailed enough to build pedigrees, so we measured heritability using the weighted regression of the median parturition date of each daughter against the median parturition date of her mother [65]. To assess whether roe deer birth date was under selection, we analyzed the relationship between birth date and early survival at the level of the individual with a generalized linear model and a logit link. We included year in the model to control for interannual variation in environmental conditions. We tested for linear, quadratic, and threshold effects of birth date on individual early survival. Finally, we tested for an interaction between birth date and year to investigate whether the selection pressure was similar over time.

### Demographic Consequences of the Mismatch

To assess whether roe deer exhibited a phenotypic response to climate change, both at the population and individual levels, we used mean cohort-specific early survival after an arcsine-square root transformation; we investigated the relationships between cohort-specific early survival and the mismatch, testing for linear, quadratic, and threshold effects of the mismatch. Each point of the regression was weighted (using the inverse of the variance of cohort-specific early survival) to account for uncertainty in the estimates of cohort-specific survival. Then, we investigated whether the mismatch was a better predictor of early survival than birth date at the individual level. We did not include year in this model as among-year variations were integrated within the mismatch variable. We tested for linear, quadratic, and threshold effects of birth date or the birth date–vegetation phenology mismatch on individual early survival with a generalized linear model and a logit link. We compared the relative fit of the different models using the Akaike Information Criterion (AIC).

To investigate the influence of plant phenology on mean fitness, we built an Integral Projection Model (IPM, [43],[44]) describing the dynamics of parturition date in the population. Selection and estimations for the models describing the four functions defining the IPM (survival, recruitment, transition, and inheritance) are detailed in the Supporting Information section. This IPM allowed us to investigate the influence of the timing of peak resource availability on the outputs of the model: the annual asymptotic population growth rate, in other words, annual mean fitness over the study period. As previous studies in this population have revealed no effect of density dependence on any of the demographic parameters, we did not include density in our demographic analysis (supplementary material of [50]).

## Supporting Information

Figure S1 **Influence of individual birth date (expressed as the day of year between 110 (20th April) and 161 (10th June)) on individual early survival in the roe deer population of Trois Fontaines, France.** The predicted values are presented after back-transformation from a logit scale with 95% confidence intervals (dashed lines). Data used to fit the model are represented by black squares whose size is proportional to the number of observed births within periods of 2 d.
(EPS)

Figure S2 **Recruitment, survival, transition, and inheritance functions in the roe deer population of Trois Fontaines, France, used to build the IPM.** Parturition date varies between the days of the year of 110 (20th April) and 161 (10th June).
(EPS)

Figure S3 **Temporal variation in mean annual reproductive success (measured in fall) of roe deer females in the population of Trois Fontaines from 1985 to 2010.** Reproductive success was estimated by direct observations of the maternal group (mother and fawns) between September and December to determine the number of fawns successfully weaned. Predicted trends are presented as black lines with 95% confidence intervals (dashed lines). The size of the circle is proportional to the number of females for which reproductive success was recorded each year.
(EPS)

Figure S4 **Annual median parturition dates in the population of roe deer from Trois Fontaines, France, from 1985 to 2011.** (A) Observed and (B) predicted (from the IPM) parturition dates. Standard errors of median observed and mean predicted parturition dates are provided.
(EPS)

Figure S5 **Stable distribution of parturition date (A) and mean parturition date in relation to age (B) predicted from the IPM in the population of roe deer from Trois Fontaines, France.** Parturition date varies between the days of the year of 91 (1st April) and 181 (30th June).
(EPS)

Table S1 **Influence of environmental variables on annual median birth date.** We investigated the influence of flowering date in the vineyards of the Champagne region (Flow. date), annual mean temperature (Annual T), mean spring (April, May, and June) temperature (Spring T), sum of spring precipitation (Spring Prec), sum of degree-days above 7°C before the birth season (SDD), mean winter (January, February, March) temperature (Winter T), sum of winter precipitation (Winter Prec), mean fall (October, November, December) temperature (Fall T), sum of fall precipitation (Fall Prec), mean summer (July and August) temperature (Summer T), and sum of summer precipitation (Summer Prec). These periods where chosen in relation to the reproductive cycle of roe deer: births occur in spring, true gestation occurs during winter, embryonic diapause occurs in fall, and the rut takes place in summer. *The correlation between median birth date and sum of degree-days was performed excluding the outlier year of 1986.
(PDF)

Table S2 **Individual early survival (recruitment function) in the roe deer population of Trois Fontaines, France.**
(PDF)

Table S3 **Transition function between two successive parturition dates in the roe deer population of Trois Fontaines, France.**
(PDF)

Table S4 **Inheritance function of parturition date between mother and daughter in the roe deer population of Trois Fontaines, France.**
(PDF)

Table S5 **Models of the functions used to build the IPM describing the distributions of parturition date (** ***PD*** **).** ***M*** **, Mother;** ***D*** **, Daughter.**
(PDF)

Table S6 **Datafile.** Year of birth, sex (1, male; 2, female), birth date (day of the year), family (0, single fawns; >0, identity of the twin pair) and early survival (0, died; 1, survived until 8 mo of age).
(XLSX)

Text S1 **Integral Projection Model on parturition date.**
(DOC)

## Abbreviations

IPM: Integral Projection Model
