# Supplementary material for: Mismatch Between Birth Date and Vegetation Phenology Slows the Demography of Roe Deer
Source: PLoS Biol. 2014 Apr 1;12(4):e1001828. doi: 10.1371/journal.pbio.1001828 (PMC3972086; doi:10.1371/journal.pbio.1001828)
Supplement: Table S1 — Influence of environmental variables on annual median birth date. We investigated the influence of flowering date in the vineyards of the Champagne region (Flow. date), annual mean temperature (Annual T), mean spring (April, May, and June) temperature (Spring T), sum of spring precipitation (Spring Prec), sum of degree-days above 7°C before the birth season (SDD), mean winter (January, February, March) temperature (Winter T), sum of winter precipitation (Winter Prec), mean fall (October, November, December) temperature (Fall T), sum of fall precipitation (Fall Prec), mean summer (July and August) temperature (Summer T), and sum of summer precipitation (Summer Prec). These periods where chosen in relation to the reproductive cycle of roe deer: births occur in spring, true gestation occurs during winter, embryonic diapause occurs in fall, and the rut takes place in summer. *The correlation between median birth date and sum of degree-days was performed excluding the outlier year of 1986. (PDF) [file pbio.1001828.s006.pdf]

Table S1: Influence of environmental variables on annual median birth date. We investigated the influence of flowering date in the vineyards of the Champagne region (Flow. date), annual mean temperature (Annual T), mean spring (April, May and June) temperature (Spring T), sum of spring precipitation (Spring Prec), sum of degree-days above 7°C before the birth season (SDD), mean winter (January, February, March) temperature (Winter T), sum of winter precipitation (Winter Prec), mean fall (October, November, December) temperature (Fall T), sum of fall precipitation (Fall Prec), mean summer (July and August) temperature (Summer T) and sum of summer precipitation (Summer Prec). These periods where chosen in relation to the reproductive cycle of roe deer: births occur in spring, true gestation occurs during winter, embryonic diapause occurs in fall and the rut takes place in summer. * The correlation between median birth date and sum of degree-days was performed excluding the outlier year of 1986.

	ρ	**t-value**	**P-value**
Flow. date	0.11	0.57	0.58
Annual T	−0.12	−0.63	0.54
Spring T	−0.07	−0.35	0.73
Spring Prec	> 0.01	−0.03	0.98
SDD*	−0.21	−1.06	0.30
Winter T	−0.04	−0.19	0.85
Winter Prec	0.27	1.39	0.18
Fall T	−0.24	−1.30	0.20
Fall Prec	−0.08	−0.42	0.68
Summer T	−0.03	−0.17	0.87
Summer Prec	−0.20	−1.04	0.31
