# Supplementary material for: Mismatch Between Birth Date and Vegetation Phenology Slows the Demography of Roe Deer
Source: PLoS Biol. 2014 Apr 1;12(4):e1001828. doi: 10.1371/journal.pbio.1001828 (PMC3972086; doi:10.1371/journal.pbio.1001828)
Supplement: Table S2 — Individual early survival (recruitment function) in the roe deer population of Trois Fontaines, France. (PDF) [file pbio.1001828.s007.pdf]

Table S2: Individual early survival (recruitment function) in the roe deer population of Trois Fontaines, France

A.

**Model**	$\boldsymbol{k}$	**ΔAIC**	$\boldsymbol{wi}$
$MisT$	4	0.000	0.690
$Mis + Mis^2$	4	1.783	0.283
Mis	3	7.682	0.015
PDT	4	8.897	0.008
$PD + PD^2$	4	9.739	0.005
PD	3	11.556	0.002
1	2	29.261	0.000

B.

**Variable**	**estimate**	**SE**
intercept	0.743	0.201
MisT	-0.048	0.009

A. Effects of maternal parturition date (PD) or the parturition date-vegetation phenology mismatch (Mis) on individual early survival of roe deer. We tested for linear, quadratic and threshold (PDT and MisT) effects of both variables. k indicates the number of estimated parameters, ΔAIC indicates the difference in the Akaike information criterion between two competing models, and wi corresponds to Akaike weights. B. Parameter estimates (on a logit scale) and their associated standard errors (SE) from the best model explaining observed variation in individual early survival.
