# Supplementary material for: Mismatch Between Birth Date and Vegetation Phenology Slows the Demography of Roe Deer
Source: PLoS Biol. 2014 Apr 1;12(4):e1001828. doi: 10.1371/journal.pbio.1001828 (PMC3972086; doi:10.1371/journal.pbio.1001828)
Supplement: Table S3 — Transition function between two successive parturition dates in the roe deer population of Trois Fontaines, France. (PDF) [file pbio.1001828.s008.pdf]

Table S3: Transition function between two successive parturition dates in the roe deer population of Trois Fontaines, France

A.

Variable	estimate	SE	t-value
intercept	89.360	15.461	5.780
PD	0.340	0.114	2.990

B.

Variable	estimate	SE	t-value
intercept	65.280	14.700	4.441

A. Effects of parturition date at time t (PD) on parturition date at time $t+1$ (mean of the transition function). B. Intercept of the squared residuals of the regression between parturition date at time $t+1$ and parturition date at time t (variance of the transition function). For each model, maternal identity was included as a random effect on the intercept. Parameter estimates and their associated standard errors (SE) and t-values are presented.
