# Supplementary material for: Mismatch Between Birth Date and Vegetation Phenology Slows the Demography of Roe Deer
Source: PLoS Biol. 2014 Apr 1;12(4):e1001828. doi: 10.1371/journal.pbio.1001828 (PMC3972086; doi:10.1371/journal.pbio.1001828)
Supplement: Table S4 — Inheritance function of parturition date between mother and daughter in the roe deer population of Trois Fontaines, France. (PDF) [file pbio.1001828.s009.pdf]

Table S4: Inheritance function of parturition date between mother and daughter in the roe deer population of Trois Fontaines, France

A.

**Variable**	**estimate**	**SE**	**t-value**
intercept	122.69	15.53	7.90
Dage	-3.17	2.51	-1.26
PD	0.15	0.10	1.46

B.

**Variable**	**estimate**	**SE**	**t-value**
intercept	42.60	47.89	0.89
Dage	2.645	25.25	0.11

A. Effects of maternal parturition date (PD) and daughter age on daughter parturition date (mean of the inheritance function). B. Effects of daughter age (Dage) on the squared residuals of the regression between daughter parturition date and mother parturition date (variance of the inheritance function). For each model, daughter age was included as a two-class factor (2y.o. vs $>$ 2y.o. females, Dage), while daughter and maternal identities were included as random effects on the intercept. Parameter estimates and their associated standard errors (SE) and t-values are presented.
