# Supplementary material for: Mismatch Between Birth Date and Vegetation Phenology Slows the Demography of Roe Deer
Source: PLoS Biol. 2014 Apr 1;12(4):e1001828. doi: 10.1371/journal.pbio.1001828 (PMC3972086; doi:10.1371/journal.pbio.1001828)
Supplement: Table S5 — Models of the functions used to build the IPM describing the distributions of parturition date ( PD ). M , Mother; D , Daughter. (PDF) [file pbio.1001828.s010.pdf]

Table S5. Models of the functions used to build the IPM describing the distributions of parturition date (PD). M: Mother, D: Daughter

**recruitment**	$R(PD) = \frac{1}{1+e^{-(0.743-0.048*(PD-annualobservedfloweringdate-mismatchin1985)))}}$
**transition**	$G(PD_{t+1}\|PD_t) = \frac{1}{\sqrt{2\pi*65.280}}e^{-\frac{PD_{t+1}-(89.360+0.340*PD_t))^2}{2*65.280}}$
**inheritance**	$I(PD_D\|PD_M) = \frac{1}{\sqrt{2\pi*(42.60+2.645)}}e^{-\frac{(PD_D-(122.69-3.17+0.15*PD_M))^2}{2*(42.60+2.645)}}$
**survival**	$S_{1y.o.} = 0.815$, $S_{2-7y.o.} = 0.924$, $S_{8-12y.o.} = 0.767$, $S_{>13y.o.} = 0.439$
