# Supplementary material for: Mismatch Between Birth Date and Vegetation Phenology Slows the Demography of Roe Deer
Source: PLoS Biol. 2014 Apr 1;12(4):e1001828. doi: 10.1371/journal.pbio.1001828 (PMC3972086; doi:10.1371/journal.pbio.1001828)
Supplement: Text S1 — Integral Projection Model on parturition date. (DOC) [file pbio.1001828.s012.doc]

**Text S1: Integral Projection Model on parturition date**

IPMs model the dynamics of a distribution of a continuous character over time [1, 2]. We used an IPM to model the distribution of female parturition dates in the roe deer population of Trois Fontaines, France. The distribution of parturition dates in the population at time $t+1$ depends on the distribution of parturition dates at time $t$ and four functions: survival, recruitment, transition between two successive parturition dates and inheritance of parturition date between mother and daughter.

We assumed that adult female survival was not influenced by either parturition date or by the mismatch between vegetation phenology and parturition date because adult roe deer survival is high and constant over years [3]. The recruitment function models the number of offspring a given female has weaned successfully at the onset of winter. As most roe deer females give birth to two fawns [4]), we modeled recruitment as individual early survival from birth to the onset of winter (at 8 months of age). The inheritance function determines the parturition date of offspring at first reproduction in relation to the parturition date of the mother. The transition function gives the probability of transition between two successive parturition dates.

For annual adult survival, we used constant values for all four age-classes: yearling (from 8 to 20 months of age), prime-aged adult (from 2 to 8 years), old (from 8 to 13 years) and senescent (beyond 13 years of age) ([3], Figure S2). The transition and the inheritance functions were assumed to have a normal probability density function with a mean and a variance modeled by two linear mixed models depending on parturition date and including maternal identity as a random effect on the intercept (Figure S2). The transition function was estimated from 79 successive pairs of parturition dates from 44 mothers. The mean of the transition function linked the parturition date at $t+1$ to the parturition date at $t$ [5]. The variance of the transition function was estimated from the squared residuals of the previous relationship and was kept constant. The mean of the inheritance function was estimated from 82 daughter-mother pairs of parturition dates from 28 different mothers. As we had repeated measures of daughter parturition dates (2.3 on average per female), we entered daughter identity in addition to mother identity as random factors on the intercept. The daughter's age for a given parturition event was entered in the model to estimate the relationship between maternal parturition date and parturition date of her 2 year-old daughter (for which only 10 data pairs were available). For the variance of the inheritance function, we proceeded in the same way as for the variance of the transition function.

To estimate the recruitment function, we performed model selection on individual early survival, from birth to 8 months of age by fitting regression models including parturition date or the parturition date-vegetation phenology mismatch as the independent variable. We tested for linear, quadratic and threshold effects of both variables. The parturition date-vegetation phenology mismatch was estimated as the difference between parturition date and the annual flowering date of vineyards in the Champagne region. We considered that yearling females did not reproduce (age at first parturition is 2 years in roe deer). We fitted generalized linear models with a logit link and a binomial distribution on 817 reproduction events. Model selection was performed using the Akaike Information Criterion [6]. AIC weights (\textit{wi}) were calculated to measure the likelihood that a candidate model is the best among the set of fitted models.

We recorded the parameters (intercepts and slope) of each function. We then constructed IPMs from these parameters (Table S5). To construct our matrix, we generated 200 parturition date classes between the 1\textsuperscript{st} of April and the 30\textsuperscript{th} of June. All statistical analyses were performed with the software R from codes (available on request from FP) based on [1] and [7]. For each year, we performed a deterministic IPM where only annual flowering date varied among years and estimated yearly asymptotic population growth rates and mean parturition date [7, 8].

**References**

1. Easterling MR, Ellner SP, Dixon PM (2000) Size-specific sensitivity: applying a new structured

population model. Ecology 81:694–708.

2. Ellner S, Rees M (2006) Integral projection models for species with complex demography. Am Nat

167:410–428.

3. Gaillard JM, et al. (1993) Roe deer survival patterns: a comparative analysis of contrasting population.

J Anim Ecol 62:778–791.

4. Gaillard JM, Andersen R, Delorme D, Linnell JDC (1998) Family effects on growth and survival of

juvenile roe deer. Ecology 78:2878–2889.

5. Plard F, et al. (2013) Parturition date for a given female is highly repeatable within five roe deer

populations. Biol Lett 9:20120841.

6. Burnham KP, Anderson DR (2002) Model selection and multimodel inference A practical information-

theoretic approach (Springer-Verlag, New York, USA, SpringerVerlag, New York), 2nd edition.

7. Coulson T, Tuljapurkar S, Childs DZ (2010) Using evolutionary demography to link life history theory,

quantitative genetics and population ecology. J Anim Ecol 79:1226–1240.

8. Coulson T (2012) Integral projections models, their construction and use in posing hypotheses in

ecology. Oikos 121:1337–1350
